# Circulating sphingolipids in heart failure

**DOI:** 10.3389/fcvm.2023.1154447

**Published:** 2023-05-09

**Authors:** Anna Kovilakath, George Wohlford, L. Ashley Cowart

**Affiliations:** ^1^ Department of Human and Molecular Genetics, Virginia Commonwealth University, Richmond, VA, United States; ^2^ Pauley Heart Center, Virginia Commonwealth University, Richmond, VA, United States; ^3^ Department of Biochemistry and Molecular Biology and the Massey Cancer Center, Virginia Commonwealth University, Richmond, VA, United States; ^4^ Richmond Veteran's Affairs Medical Center, Richmond, VA, United States

**Keywords:** sphingolipid, ceramide, heart failure, HFrEF—heart failure with reduced ejection fraction, HFpEF—heart failure with preserved ejection fraction, serine palmitoyltransferase, lipidomics, ceramide score

## Abstract

Lack of significant advancements in early detection and treatment of heart failure have precipitated the need for discovery of novel biomarkers and therapeutic targets. Over the past decade, circulating sphingolipids have elicited promising results as biomarkers that premonish adverse cardiac events. Additionally, compelling evidence directly ties sphingolipids to these events in patients with incident heart failure. This review aims to summarize the current literature on circulating sphingolipids in both human cohorts and animal models of heart failure. The goal is to provide direction and focus for future mechanistic studies in heart failure, as well as pave the way for the development of new sphingolipid biomarkers.

## Introduction

Heart failure (HF) is a debilitating medical condition where the heart is unable to meet the metabolic demands of the body. It is a widespread clinical syndrome affecting at least 64 million people globally, and this number is expected to rise (1, 2). HF poses a significant economic and clinical burden and dramatically impacts patients' quality of life (3). Different classifications of HF exist, with the most widely accepted based on the percentage of blood volume ejected by the left ventricle (LV) during systole, i.e., heart failure with reduced ejection fraction (HFrEF, LVEF ≤40%), heart failure with preserved ejection fraction (HFpEF, LVEF ≥50%), and as of 2016, heart failure with borderline/mildly reduced ejection fraction (HFbEF/HFmrEF, LVEF 41%–49%) (4–6). Though, there is some controversy related to the cut-off points in this HF classification. Currently, no human studies link HFbEF/HFmrEF with sphingolipids and will not be discussed further in this review.

Advances in medicine over the past decade have improved the quality of life and outcomes for patients with HFrEF. These include pharmacotherapies such as Aldosterone/Mineralocorticoid Receptor Antagonists (ARA/MRA, e.g., spironolactone), Angiotensin Receptor-Neprilysin Inhibitor (ARNI, e.g., Sacubitril-valsartan), ventricular assist devices (VAD), cardiac resynchronization therapy (CRT), and implantable cardioverter-defibrillator (ICDs) (4, 7–10). However, HFpEF has now become the most prevalent form of HF, and clinical trials of HFpEF pharmacotherapies, such as Sodium-GLucose co-Transporter 2 (SGLT2) inhibitors (e.g., empagliflozin), ARNI, and ARA/MRA, and novel device therapies like InterAtrial Shunt Devices (IASD), have demonstrated only modest reductions in the risk of hospitalization (4, 9, 11–16). This is likely due to the highly heterogenous nature of the HFpEF population with respect to pathogenesis, pathophysiology, and non-cardiac comorbidities. Despite these advancements, the median 5-year mortality rates upon clinical diagnosis of HFrEF and HFpEF are 75.3% and 75.7%, respectively (17).

**Figure 1:**
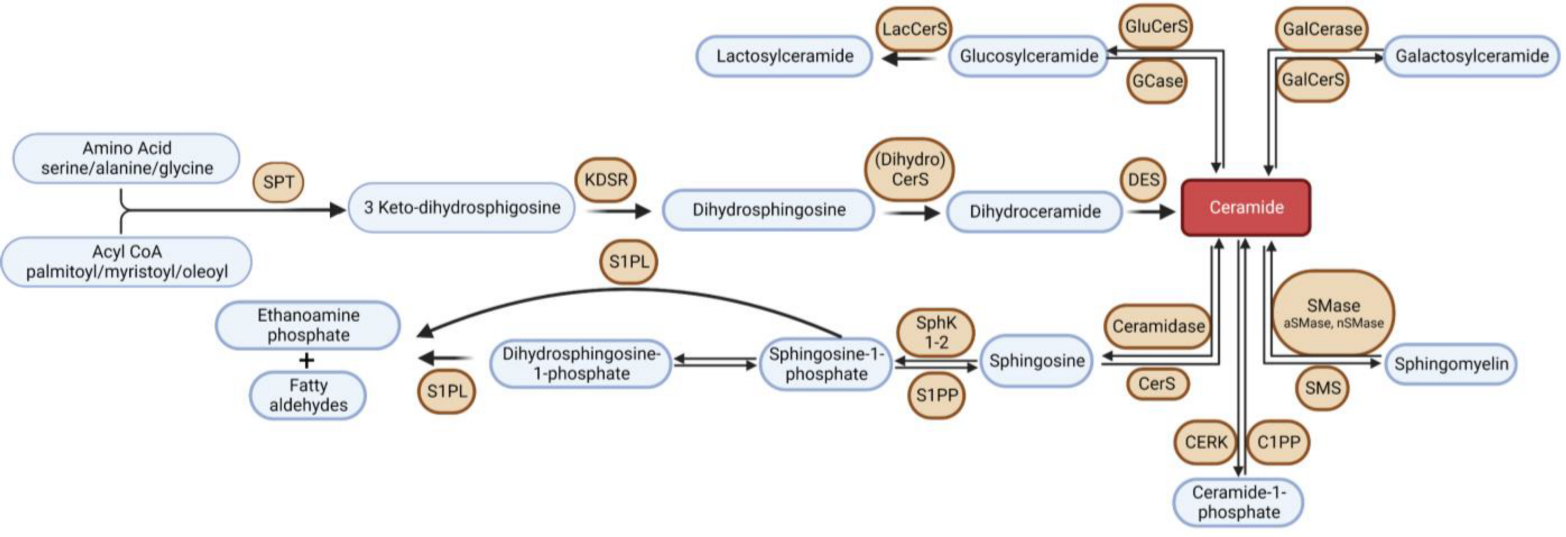
Overview of sphingolipid metabolism. SPT, serine palmitoyltransferase; KDSR, 3-keto-dihydrosphingosine reductase; DES, dihydroceramide desaturase; GCase, glucocerebrosidase; GluCerS; glucosylceramide synthase; LacCerS, lactosylceramide synthase; GalCerS, galactosylceramide synthase; SMase, sphingomyelinase; SMS, sphingomyelin synthase; CerS, ceramide synthase; C1PP, ceramide-1-phosphate phosphatase; CERK,; S1PP, sphingosine-1-phosphate phosphatase; SphK, sphingosine kinase; S1PL, sphingosine-1-phosphate lyase.

Sphingolipids are a class of lipids, conserved across all eukaryotic organisms, characterized by an amino alcohol headgroup and fatty acid carbon chain. Though sphingolipids serve essential structural roles in membranes, many sphingolipid metabolites are considered bioactive lipids and play a central roles in inflammation, autophagy, apoptosis, immune cell trafficking, cell survival, metabolism, mitochondrial function, and many other critical cell processes (18, 19). Disrupted sphingolipid metabolism has been implicated in HF pathophysiology, and thus, enzymes involved in sphingolipid metabolism (or the protein signaling effectors) have become potential therapeutic targets. Consequently, over the past 15 years, there has been a surge of interest in the study of sphingolipid metabolism in HF (19). Sphingolipids are synthesized *de novo* in the endoplasmic reticulum *via* condensation of an amino acid, typically serine, and a fatty acid, catalyzed by serine palmitoyltransferase (SPT) (20). This pathway is depicted in Figure 1. This reaction produces a sphingoid base, the molecular scaffold on which all sphingolipids are constructed. SPT exists as a heterooligomeric enzyme consisting of a dimer of tetramers, with SPTLC1 being essential, and SPTLC2 or 3 required for catalytic activity. Two small subunits, SPTssa and SPTssb, differentially regulate Acyl-CoA chain length utilization by SPT1 and 2/3 (21). A fourth subunit, ORMDL, acts as three isoforms (ORMDLs 1–3) to sense membrane sphingolipids to attenuate sphingolipid synthesis in the presence of high membrane ceramide (Cer) (22). Differences between these three isoforms, ORMDL 1, 2, and 3, are very poorly understood at present. Nonetheless, the composition of the SPT complex determines Acyl-CoA selectivity and thus determines the catalytic product. For example, when the subunits SPTLC1/SPTLC2/SPTssa are present in SPT, then palmitoyl-CoA is condensed with an amino acid to synthesize all downstream sphingolipid species with an 18-carbon sphingoid backbone, i.e., d18 (23). This generates the canonical sphingoid bases that have been studied for over 150 years. Alternatively, SPTLC3 can substitute for SPTLC2, and SPTssb can substitute for SPTssa, leading to production of sphingolipid species with alternative carbon sphingoid backbones (e.g., d16, and d20) (24–26). The complexity of SPT and the variety of sphingoid bases has only recently become appreciated (largely driven by the discovery of SPTLC3 and the small subunits). Recent research has shown that the SPT enzyme and the sphingoid bases it produces are crucial components of cardiovascular health, with several studies supporting their relevance (27–30). For example, we previously demonstrated in mice hearts where SPTLC3 is expressed, the SPT complex can generate a d16-derived sphingolipid from myristoyl-CoA, which was shown to stimulate cardiomyocyte apoptosis (25).

**Figure 2:**
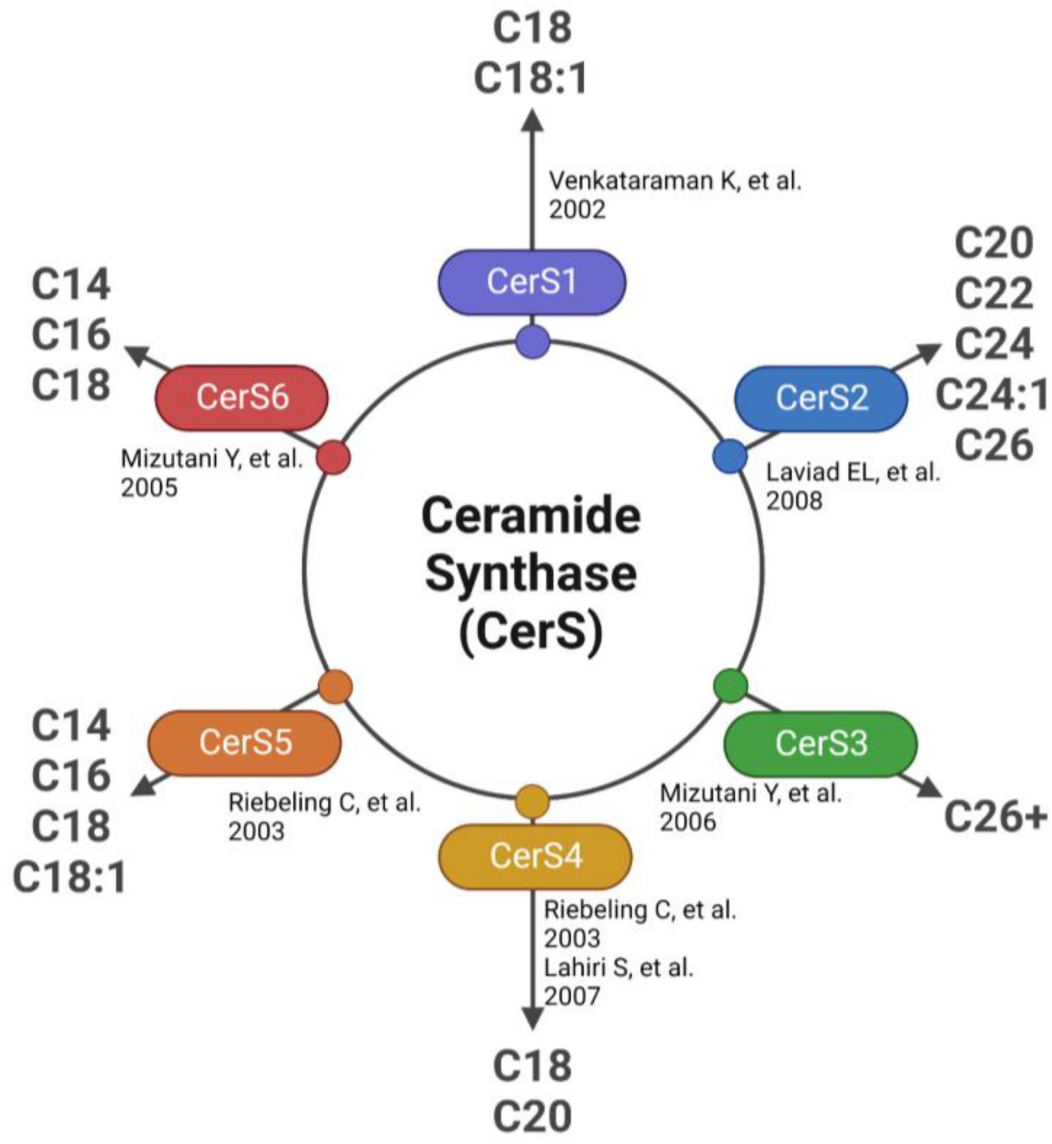
Ceramide synthase (CerS) substrate selectivity.

The first detectable sphingolipid to be synthesized is the sphingoid base, dihydrosphingosine (DHS), which is then N-acetylated by one of 6 isoforms of (dihydro) ceramide synthase (CerS), with different chain length fatty acids to synthesize dihydroceramides (DHC) (31). CerS1 typically adds 18-carbon acyl chain length (C18:0, and C18:1); CerS2 adds C22:0, C24:0, C24:1, C26:0 and C26:1; CerS3 adds C22:0 to C36:0; CerS4 adds C18:0 to C22:0; CerS5 adds C14:0 to C18:0; and CerS6 adds C14:0 to C18:0 (Figure 2). The acyl chain length ranges from medium MCFA (C12–14), long LCFA (C16–20), very long VLCFA (C22–26), and ultra-long chain fatty acids ULCFA (>C26) (32–38). DHC desaturase then converts DHC to Cer, the backbone of all complex sphingolipids (39). Acid or alkaline ceramidase enzymes (AC or ACER 1–3) can then hydrolyze Cer to yield sphingosine (Sph), which can be phosphorylated by Sph kinase (SphK) 1 or 2 to generate sphingosine-1-phosphate (S1P) or dihydrosphingosine-1-phosphate (DHS1P) (Figure 1). S1P is a potent signaling molecule that can bind to one of its five G protein-coupled receptors (S1PR 1–5) to elicit downstream effector cell activity or be exported extracellularly by Spinster 2 (Spns2). Sph can also be “salvaged” and re-incorporated into Cer through the action of CerS enzymes. This mechanism allows cells to “remodel” Cer pools. If Cer is not hydrolyzed, it can be further metabolized into sphingomyelin (SM) or glycosphingolipids (GSL) through the addition of various headgroups. SM can then be hydrolyzed by acid or neutral sphingomyelinase (aSMase, nSMase) to yield Cer. GSLs containing Cer and glucose are the predominant precursors of globosides and gangliosides, while those with Cer and galactose synthesize downstream sulfatides. To exit the sphingolipid *de novo* pathway, S1P lyase (S1PL) catabolizes S1P or DHS1P in lysosomes and plasma membranes to yield a fatty aldehyde and phosphoethanolamine (40). The heart requires a constant supply of energy to maintain its contractile function, which is primarily provided through fatty acid oxidation (41). Studies have shown that disruptions in sphingolipid metabolism contribute to impaired fatty acid oxidation, with the Cer-S1P rheostat playing a particularly important role (42). The dogma being Cer accumulation is harmful, while S1P accumulation is beneficial (42–45). Therefore, interconversion between these sphingolipid species is a highly regulated process, and any small deviation in anabolism, catabolism or substrate availability can lead to abnormal accumulation of one or more sphingolipid species, resulting in dysregulated fatty acid supply in the heart (46). As such, alterations in sphingolipid content and profiles have become a key area of investigation in HF research.

In this review, we examine the relationship between sphingolipids and HF in both human study cohorts and animal models. We analyze the current literature to identify potential biomarkers and druggable targets for the detection and treatment of HF.

## Sphingolipids in heart failure

Many studies, such as the Framingham Heart Study (FHS), have been ongoing for decades and have analyzed traditional risk factors like obesity, smoking status, and LDL-cholesterol from these patient cohorts. However, only in recent years have investigators started to address circulating sphingolipids in subjects from these studies through *post hoc* analyses. This involves mass spectrometry to identify and quantify sphingolipids in blood serum, blood plasma, or myocardial tissue. This method yields measurements for numerous sphingolipids, which can then be correlated to disease and other clinical parameters to gain a better understanding of their relationship. Current literature on sphingolipid associations with HF can broadly be divided into two types: sphingolipid associations in patients with incident HF (summarized in Table 1), and secondary endpoints in patients with prevalent HF (summarized in Table 2).

**Table 1:** Studies on sphingolipids associated with incident heart failure.

Study Name, Year Published, Reference(s)	Study Design	*N* total	Outcome	Sample Type, Sphingolipid Species	HR/RR	95% CI	*P*-value
Framingham Heart Study (FHS), 2018, (47)	Longitudinal, Community-based, Cohort	2,542	Incident HF	Plasma, i. Cer C16:0 ii. Cer C24:0 iii. Cer (C22:0/C16:0) iv. Cer (C24:0/C16:0)	i. 1.16 ii. 0.75 iii. 0.70 iv. 0.66	i. 0.89, 1.50 ii. 0.57, 1.00 iii. 0.57, 0.87 iv. 0.51, 0.82	i. ns ii. * iii. ** iv. ***
Study of Health in Pomerania (SHIP), 2018, (47)	Longitudinal, Community-based, Cohort	1,935	Incident HF	Plasma, i. Cer C16:0 ii. Cer C24:0 iii. Cer (C22:0/C16:0) iv. Cer (C24:0/C16:0)	i. 1.12 ii. 1.04 iii. 0.98 iv. 0.92	i. 0.96, 1.31 ii. 0.86, 1.25 iii. 0.81, 1.18 iv. 0.75, 1.13	i. ns ii. ns iii. ns iv. ns
Meta analysis of Framingham Heart Study (FHS), And Study of Health in Pomerania (SHIP), 2018 (47)	Longitudinal, Community-based, Cohort	2,542 + 1,935	Incident HF	Plasma, i. Cer C16:0 ii. Cer C24:0 iii. Cer (C22:0/C16:0) iv. Cer (C24:0/C16:0)	i. 1.13 ii. 0.92 iii. 0.83 iv. 0.78	i. 0.98, 1.31 ii. 0.74, 1.13 iii. 0.67, 1.05 iv. 0.61, 1.00	i. ns ii. ns iii. ns iv. *
Cardiovascular Health Study (CHS), 2019^@@^, (48)	Longitudinal, Population-based, Cohort	4,249	Incident HF 1. Total HF	Plasma, 1. Total HF i. Cer C16:0 ii. Cer C20:0 iii. Cer C22:0 iv. Cer C24:0 v. SM C16:0 vi. SM C20:0 vii. SM C22:0 viii. SM C24:0	1. Total HF i. 1.25 ii. 0.94 iii. 0.85 iv. 0.94 v. 1.28 vi. 0.83 vii. 0.81 viii. 0.83	1. Total HF i. 1.16, 1.36 ii. 0.87, 1.01 iii. 0.78, 0.92 iv. 0.87, 1.02 v. 1.18, 1.40 vi. 0.77, 0.90 vii. 0.75, 0.88 viii. 0.77, 0.90	1. i. *** ii. ns iii. *** iv. ns v. *** vi. *** vii. *** viii. ***
			2. HFpEF	Plasma, 2. HFpEF i. Cer C16:0 ii. Cer C20:0 iii. Cer C22:0 iv. Cer C24:0 v. SM C16:0 vi. SM C20:0 vii. SM C22:0 viii. SM C24:0	2. HFpEF i. 1.25 ii. 1.01 iii. 0.93 iv. 0.98 v. 1.19 vi. 0.88 vii. 0.94 viii. 0.93	2. HFpEF i. 1.11, 1.40 ii. 0.91, 1.13 iii. 0.83, 1.05 iv. 0.87, 1.10 v. 1.06, 1.35 vi. 0.79, 0.99 vii. 0.83, 1.06 viii. 0.83, 1.05	2. i. *** ii. ns iii. ns iv. ns v. ** vi. * vii. ns viii. ns
			3. HFrEF	Plasma, 3. HFrEF i. Cer C16:0 ii. Cer C20:0 iii. Cer C22:0 iv. Cer C24:0 v. SM C16:0 vi. SM C20:0 vii. SM C22:0 viii. SM C24:0	3. HFrEF i. 1.23 ii. 1.01 iii. 0.87 iv. 0.89 v. 1.11 vi. 0.91 vii. 0.88 viii. 0.89	3. HFrEF i. 1.07, 1.42 ii. 0.89, 1.16 iii. 0.75, 1.01 iv. 0.78, 1.02 v. 0.96, 1.28 vi. 0.80, 1.04 vii. 0.76, 1.02 viii. 0.77, 1.03	3. i. ** ii. ns iii. ns iv. ns v. ns vi. ns vii. ns viii. ns
Strong Heart Family Study (SHFS)^##^, 2022, (49)	Longitudinal, Population-based, (Nested) Case-control	33	Incident HF	Plasma, i. Cer C16:0 ii. Cer C20:0 iii. Cer C22:0 iv. Cer C24:0 v. SM C16:0 vi. SM C20:0 vii. SM C22:0 viii. SM C24:0	i. 2.57 ii. 0.83 iii. 0.67 iv. 0.45 v. 0.69 vi. 1.27 vii. 1.59 viii. 1.52	i. 0.76, 8.66 ii. 0.32, 2.18 iii. 0.26, 1.72 iv. 0.14, 1.44 v. 0.13, 3.74 vi. 0.38, 4.22 vii. 0.41, 6.10 viii. 0.49, 4.77	i. ns ii. ns iii. ns iv. ns v. ns vi. ns vii. ns viii. ns
Prevención con Dieta Mediterránea (PREDIMED)^%%^, 2020, (50)	Nutritional Intervention, Randomized, Nested Case-control	838 (331 HF + 507 control)	Incident HF	Plasma, i. Cer C16:0 ii. Cer C24:1 iii. SM C16:1 iv. SM C18:0 v. SM C18:1 vi. SM C24:0 vii. SM C24:1 viii. Sph	i. 1.27 ii. 0.86 iii. 1.85 iv. 1.43 v. 0.66 vi. 0.70 vii. 0.88 viii. 1.14	i. 1.06, 1.51 ii. 0.703, 1.00 iii. 1.32, 2.60 iv. 1.01, 2.02 v. 0.43, 1.02 vi. 0.57, 0.85 vii. 0.71, 1.09 viii. 1.00, 1.31	i. * ii. * iii. *** iv. * v. ns vi. *** vii. ns viii. ns
European Investigation into Cancer (EPIC)-Potsdam^%%^, 2020, (50)	Longitudinal, Population-based, Case-control	2,414 (87 HF + 2,327 at-risk)	Incident HF	Plasma, i. Cer C16:0 ii. Cer C24:1 iii. SM C18:0 iv. SM C18:1 v. SM C24:1 vi. SM C24:0	i. 1.72 ii. 0.92 iii. 1.54 iv. 0.63 v. 1.27 vi. 0.68	i. 1.14, 2.60 ii. 0.62, 1.38 iii. 0.82, 2.90 iv. 0.34, 1.15 v. 0.88, 1.83 vi. 0.44, 1.04	i. ** ii. ns iii. ns iv. ns v. – vi. ns
Poland Bialystok HF Study, 2012, (51)	Longitudinal, Population-based, Case-control	62 (47 HFrEF + 15 control)	Incident HF	Plasma, i. *Total Cer* ii. S1P iii. DHS1P	i. – ii. – iii. –	i. – ii. – iii. –	i. ns ii. ns iii. ns
Health Research Institute Hospital La Fe (IIS La Fe) HF Study, 2022, (52)	Longitudinal, Population-based, Case-control	41 (36 HF + 5 control)	Incident HF	Myocardial Tissue, i. *Total Cer* ii. S1P	i. – ii. –	i. – ii. –	i. ** ii. *
Columbia University Medical Center HF Study, 2017, (53)		86 (64 HF + 22 control)	Incident HF	Serum, i. *Total Cer* ii. Cer C14:0 iii. Cer C16:1 iv. Cer C16:0 v. Cer C18:1 vi. Cer C18:0 vii. Cer C20:1 viii. Cer C20:0 ix. Cer C22:0 x. Cer C22:1 xi. Cer C24:1 xii. Cer C24:0	i. – ii. – iii. – iv. 1.35 v. – vi. 1.35 vii. 1.35 viii. – ix. – x. – xi. –	i. *–* ii. *–* iii. – iv. 1.13, 1.63 v. – vi. 1.13, 1.63 vii. 1.13, 1.63 viii. – ix. – x. – xi. –	i. ns ii. ns iii. ns iv. * v. ns vi. ** vii. * viii. ** ix. ns x. * xi. *** xii. *

HR, Hazard Ratio; RR, Risk Ratio; CI, Confidence Interval; SD, Standard Deviation; Cer, Ceramide; SM, Sphingomyelin; HFpEF, heart failure with preserved ejection fraction; HFrEF, heart failure with reduced ejection fraction; Sph, Sphingosine; S1P, Sphingosine-1-phosphate; DHS1P, Dihydrosphingosine-1-phosphate; ns, not significant.

* *P* < 0.05.

** *P* < 0.005.

*** *P* < 0.0001.

^@@^Multi + Cer-22 or Multi + Cer-16 model used.

^##^ Sphingolipid adjusted model used.

^%%^ Model 2 used.

**Table 2:** Studies on sphingolipids associated with secondary endpoints in patients with prevalent HF.

Study Name, Year Published, Reference(s)	Study Design	N total	Outcome	Sample Type Sphingolipid Species	HR/RR	95% CI	*P*-value
Treatment of Preserved Cardiac Function Heart Failure with an Aldosterone Antagonist (TOPCAT)^^^^, 2020, (54)	Double-blind, Randomized, Placebo-controlled	433	DHFA	Serum, i. Cer C16:0 ii. Cer C18:0 iii. Cer C18:1 iv. Cer C20:0 v. Cer 24:0 vi. Cer C24:1 vii. MHC C16:0 viii. MHC C24:1 ix. SM C16:0 x. SM C22:0	i. 1.35 ii. 1.30 iii. 1.30 iv. 1.25 v. 1.35 vi. 1.20 vii. 1.25 viii. 1.20 ix. 1.20 x. 1.20	i. 1.13. 1.63 ii. – iii. – iv. – v. 1.12, 1.61 vi. – vii. – vii. – viii. –	i. ** ii. * iii. * iv. * v. ** vi. * vii. * viii. * ix. * x. *
Gruppo Italiano per lo Studio della Sopravvivenza nella Insufficienza Cardiaca-Heart Failure (GISSI-HF), 2020, (55)	Longitudinal, Population-based, Case-control	400 (200 survivors + 200 dead)	1. HF survival	Plasma, 1. HF survival i. Cer C18:0 ii. Cer C20:0 iii. Cer C22:0 iv. Cer C24:0 v. Cer C24:1 vi. Cer (C16:0/C24:0) vii. Cer (C18:0/C24:0) viii. Cer (C20:0/C24:0) ix. Cer (C22:0/C24:0) x. Cer (C24:1/C24:0)	i. 0.09 ii. 0.08 iii. 0.53 iv. 2.27 v. 0.79 vi. 0.08 vii. 0.04 viii. 0.04 ix. 0.23 x. 0.36	i. 0.07, 0.12 ii. 0.06, 0.10 iii. 0.42, 0.63 iv. 1.78, 2.62 v. 0.63, 0.96 vi. 0.06, 0.10 vii. 0.03, 0.06 viii. 0.03, 0.042 ix. 0.21, 0.26 x. 0.29, 0.44	i. ns ii. ns iii. * iv. *** v. * vi. *** vii. ** viii. *** ix. ** x. ***
			2. HF mortality	Plasma, 2. HF mortality i. Cer C18:0 ii. Cer C20:0 iii. Cer C22:0 iv. Cer C24:0 v. Cer C24:1 vi. Cer (C16:0/C24:0) vii. Cer (C18:0/C24:0) viii. Cer (C20:0/C24:0) ix. Cer (C22:0/C24:0) x. Cer (C24:1/C24:0)	i. 0.09 ii. 0.08 iii. 0.47 iv. 1.93 v. 0.83 vi. 0.10 vii. 0.05 viii. 0.4 ix. 0.24 x. 0.45	i. 0.07, 0.12 ii. 0.07, 0.10 iii. 0.37, 0.61 iv. 1.51, 2.48 v. 0.69, 1.02 vi. 0.07, 0.13 vii. 0.04, 0.07 viii. 0.04, 0.05 ix. 0.23, 0.27 x. 0.36, 0.57	i. ns ii. ns iii. * iv. *** v. * vi. *** vii. * viii. *** ix. ** x. ***
Columbia University Medical Center HF Study, 2017, (53)		22 (7 control + 15 HF with VAD)	VAD replacement	Myocardial tissue, i. Total Cer ii. Cer C14:0 iii. Cer C16:1 iv. Cer C16:0 v. Cer C18:1 vi. Cer C18:0 vii. Cer C20:0 viii. Cer C22:1 ix. Cer C22:0 x. Cer C24:1 xi. Cer C24:0	i. – ii. – iii. 1.35 iv. – v. – vi. – vii. – viii. – ix. – x. – xi. –	i. – ii. – iii. 1.13, 1.63 iv. – v. – vi. – vii. – viii. – ix. – x. – xi. –	i. * ii. ns iii. * iv. * v. ns vi. ns vii. ns viii. ns ix. ns x. * xi. ns
Xiangya Hospital HF study, 2015, (56)	Longitudinal, Population-based, Cohort	423	HF mortality	*Total Cer* (continuous variable)	1.30	1.15–1.46	**
				*Total Cer* (≥6.05 ng/ml vs. <6.05 ng/ml)	2.07	1.53–2.81	**

HR, hazard Ratio; RR, risk ratio; CI, confidence interval; SD, standard deviation; Cer, ceramide; MHC, monohexosylceramide; SM, sphingomyelin; ns, not significant; DHFA, death or heart failure admission; VAD, ventricle assist device.

* *P* < 0.05.

** *P* < 0.005.

*** *P* < 0.0001.

^^^^ MAGGIC (Meta-Analysis Global Group in Chronic Heart Failure) model used.

Sphingolipids associated with incident heart failure are discussed in this section. Meta-analysis of PREDIMED and EPIC-Potsdam cohorts showed that Cer C16:0 was significantly increased but also ranked as one of the topmost lipid cluster networks associated with HF (50). A 2012 study by Knapp et al., found no significant association of plasma S1P, DHS1P or *total Cer* in HFrEF and HFpEF patients (51). However, the plasma of both HFpEF and HFrEF patients was characterized by significantly lower levels of free Sph and DHS, but stable S1P levels. This observation suggests that diminished action of S1P does not contribute to cardiac dysfunction in patients with chronic HF (51). A small yet more recent study by Pérez-Carrillo et al., showed that myocardial tissue from HFrEF and HFpEF patients had double S1P and triple *total Cer* levels compared to controls (52). After mRNA-sequencing, 12 sphingolipid-specific genes were differentially expressed. SPTssa, SPTssb, SPTLC1, and SPTLC3 genes were downregulated suggesting *de novo* sphingolipid synthesis is reduced. However, CerS1, which can act both *de novo* and in the salvage pathway, was upregulated, potentially explaining the increased Cer. Additionally, S1P phosphatase (S1PP), the enzyme converting S1P to Sph, and S1PR3 were downregulated in HF patients. Though this should result in decreased S1P in myocardial tissue and not the observed increase in levels (52). While this data is interesting and suggests S1P levels are not indicative of HF status, they do not provide the full picture of sphingolipid *de novo* synthesis dynamics and leave us with more questions instead of answers. A 2019 study, using the Cardiovascular Health Study (CHS) cohort, identified higher levels of SM C16:0, and Cer C16:0 associated with higher risk and SM C20:0, C22:0, C24:0, and Cer C22:0 with lower risk of developing HF, independently. Interestingly, these species had similar associations regardless of whether the patient had HFpEF or HFrEF (48).

In contrast to studies that assessed the correlation of individual sphingolipid species level with HF, some more recent studies have analyzed ratios between specific sphingolipids. Analysis of sphingolipid ratios may not only be more effective in predicting incident HF, but also adverse cardiac events. This sphingolipid ratio score is more reliable as it removes complications that arise from altered sphingolipid concentrations postprandially and/or with hyperlipidemia. Newly developed high throughput assays to quantify ratios of VLCFA/LCFA Cer or vice versa in the plasma were applied to the FHS and SHIP (Study of Health in Pomerania) studies. These assays were able to show a higher ratio in plasma Cer(C24:0/C16:0) was inversely associated with incident HF (47). Outcomes from these studies paved the way for the launch of the MI-Heart Ceramide Risk Score (CERAM) blood test used by Mayo Clinic to predict unfavorable cardiovascular events in patients (57). The test measures concentrations of plasma Cer C16:0, C18:0, C24:1 and the plasma ratios Cer(C16:0/24:0), Cer(18:0/24:0), and Cer(24:1/24:0). The risks conferred by Cer ratios are independent of sex, age, gender, LDL and other traditional factors, and continually outperform cholesterol testing.

Sphingolipids associated with secondary endpoints in patients with prevalent HF such as major adverse cardiac event(s), Ventricle Assist Device (VAD) placement or replacement, death, or heart failure admission (DHFA), and HF-related mortality are described in this section. A 2015 study by Yu et al., concluded that plasma from patients with HF who died had *total Cer* >6.05 ng/ml, and HF patients that survived (at least up to the 4 years during the study) had circulating *total Cer* <6.05 ng/ml (56). There has been no follow-up study to verify whether this or another *total Cer* threshold can predict survival in HF patients. A 2017 study from Christian Schulze's lab revealed that hearts from advanced HF patients showed significantly increased *total Cer* driven by increased Cer C16:0, C16:1 and C24:1 and increased SPTLC2 protein expression (irreconcilably no change in CerS1, CerS2 or CerS5 expression was noted) undergoing placement of VAD (53). Interestingly, there was no significant change in circulating *total Cer*, but significant increases in circulating Cer C16:0, C18:0, C20:1, C20:0, C22:1, C24:1, and C24:0. Though, after VAD implantation, these changes showed partial reversibility in myocardial tissue but not circulating Cer (53). A study by Javaheri et al., linked increased serum sphingolipids Cer C16:0 and C18:0 with death or HF admission (DHFA) in a TOPCAT study of 433 HFpEF patients (54). In a study with Italian HF patients, associated increased plasma ratios of Cer(C16:0/C24:0), Cer(C18:0/C24:0), Cer(C18:0/C24:0), Cer(C22:0/C24:0), and Cer(C24:1/C24:0), along with higher Cer C16:0, C18:0, C20:0, C22:0, and Cer C24:1 individually with increased cardiovascular mortality in ambulatory patients with chronic HF. However, these Cer to HF associations became non-significant after adjustment for established cardiovascular risk factors, medication use, and plasma N-terminal pro b-type natriuretic peptide (NT-proBNP) concentrations (55). Though not a study of secondary outcomes in patients with prevalent HF, A subset of the major findings in the 2018 study using the community-based cohorts, FHS and SHIP, also showed that Cer C16:0 is inversely correlated while Cer C24:0, Cer(C22:0/C16:0), and Cer(C24:0/C16:0) were positively correlated with predictive information about CVD and all-cause mortality in the general population 6 years before the actual onset of disease (47).

Similar approaches have been used not in incident HF populations but in populations presenting with pre-HF etiologies such as type two diabetes (T2D), obesity or incident coronary heart disease (CHD), including but not limited to myocardial infarction (MI), atrial fibrillation, coronary insufficiency, adverse cardiac remodeling, and angina pectoris. The SHS (Strong Heart Study) and SHFS (Strong Heart Family Study) fare population-based longitudinal studies addressed cardiovascular disease (CVD) in several American Indian communities in Arizona, North and South Dakota, and Oklahoma. It is interesting to note that increased Cer C16:0, though not significant, correlated with increased CVD risk after diabetes onset, while the inverse was true of increased SM C22:0, C24:1, C24:0, C26:1, and SM C26:0 (49). A study by Fretts et al., using the SHS and SHFS plasma samples, showed that higher Cer C18:0, C20:0, and Cer C22:0 were associated with higher risk of diabetes (58). Mikhalkova et al., sought to determine whether there were changes in circulating Cer and circulating SM species in women with obesity and HFpEF before and after bariatric surgery. After the surgery-induced weight loss the patients showed improved symptoms, reverse cardiac and remodeling and improved relaxation. Though the weight loss was associated with reduced plasma SM C23:1, cardiac function improvement was not associated with sphingolipidomic changes, which may have been due to the relatively small sample size (*N* = 12) (59). Another study, determined that Cer C18:0, but not Cer C16:0, C24:0, or C24:1 were associated with incident of major adverse cardiovascular events (MACE), and showed stronger correlation for recurrent and fatal events than for first events (60). In contrast to the MACE study, plasma concentrations of 74 ischemic heart disease patients showed no association of Cer C16:0 Cer with LVEF. However, *total SM* and S1P were significantly lower in patients with HFrEF compared to patients with HFpEF (61). The LUdwigshafen RIsk and Cardiovascular Health (LURIC) study established well defined phenotypes for CVD, metabolic disorders, and their progression to cardiovascular complications including HF (62). Independent of traditional risk factors, SM C23:0, and SM C24:0 were the most protective sphingolipid species, intermediate protection conferred by Cer C23:0, Cer C24:0, SM C16:0, and SM C24:1, while Cer C16:0, and Cer C24:1 had the strongest positive association with CVD and mortality (63). Another study put diabetic patients on diets rich with either LCFA or MCFA. Patients on the MCFA diet showed improved systolic function with concomitant decrease of circulating sphingolipids, while subjects on the LCFA diet showed reduced stroke volume, cardiac output, and no change in systolic function which was associated with increased SM C15:0, and SM C22:1 (64). Newly developed high throughput assays to quantify ratios of VLCFA/LCFA Cer in the plasma were applied to the Framingham Heart Study and the SHIP (Study of Health in Pomerania). These assays were able to show a higher ratio in plasma Cer(C24:0/C16:0) was inversely associated with incident HF (47). A more recent study of the FHS cohort associated higher plasma Cer(C16:0/C24:0) with detrimental cardiac structural and functional changes that can lead to HF (65). A 2020 study analyzing the CHS cohort, determined that Cer C20:0, Cer C22:0, and Cer C24:0 were associated with reduced atrial fibrillation risk (66).

Taken together, these studies suggest the dogma implicating *total Cer* accumulation in HF and HF-related outcomes, holds true. Though these results are from vastly different studies, they consistently show inverse association of the LCFA Cer C16:0, synthesized by CerS5 or CerS6, and the unsaturated VLCFA Cer C24:1, synthesized by CerS2, to HF, regardless of HF classification and population ethnicity. While the saturated VLCFA Cer C24:0 and in some studies the SM C24:0 is positively correlated with HF. Though, ratios of sphingolipid LCFA/VLCFA seem to be better prognostic markers of adverse cardiac events, HF and prediction of HF-mortality up to 5 years.

## Insights from Mendelian randomization analysis studies

Developing new pharmacotherapies is a costly and time-consuming process, and drug companies are incentivized to speed up the path to market approval due to the time-limits on drug patents. However, testing agents that may augment a specific disease biomarker without fully understanding their potential mechanistic roles has led to failed clinical trials. To increase the likelihood of identifying causal biomarker-disease associations, researchers have started to employ Mendelian randomization (MR). This approach involves a genome-wide association study (GWAS) analysis to identify candidate genetic variants associated with variations in the biomarker, which are then compared to those associated with differences in the population. When a biomarker is identified and a causal effect is confirmed by MR, the probability of clinical trial success for interventions targeting that biomarker increases significantly (67).

Although MR analyses have not been reported in the literature for the association between circulating sphingolipids and HF, a GWAS analysis has been conducted for circulating sphingolipids associated with a reduction in HF events. MR analyses for sphingolipids linked to incident coronary heart disease (CHD) have also been reported, which is relevant since CHD and HF can coexist in the same patient, and patients with CHD are at increased risk for development of HF (4, 68, 69). Inclusion of these investigations in this focused review is justified given the robustness of the MR methodology, the lack of direct evidence reported in the literature for HF and sphingolipids tested with the MR methodology and the proximity of CHD to HF across the landscape of cardiovascular disease.

VLCFA Cer measured in the plasma, has been shown to be prognostic for incident HF events (relative risk of 0.75 for every 3 unit increase in plasma Cer C24:0 for HF) despite traditionally being implicated in the etiology of T2DM (30). Analyses of patient samples obtained from the FHS identified 19 genetic variants associated with lower levels of Cer C22:0 and 9 variants associated with lower levels of Cer C24:0. These 28 variants were located on chromosome 20, near the SPTLC3 encoding gene. The identified lead variant (rs4814175) was associated with 3% lower Cer C22:0 and 10% lower Cer C24:0 concentrations, though, MR analysis for this variant was not conducted (30).

The findings from another GWAS utilizing data from two different large epidemiologic datasets (*N* = 1,094 and *N* = 4,034), were congruent with the FHS findings. Variants near SPTLC3 were confirmed to be contributing factors in the variations of circulating Cer linked to CVD and T2DM (70). However, after MR analysis was performed, the lead SNP (rs680379) tested was different than what was previously identified. The new variant was used to assess the risk for incident T2DM and the resulting change in risk for incident CVD for individuals that carried the SNP. The investigators also linked circulating Cer C16:0 and DHC C22:2 with an increased risk of incident CVD in these separate patient populations. However, subsequent GWAS and MR analysis for the assessment of the association with CVD or HF events was not completed for these Cer. Reported in 2014, investigators from Sweden pooled samples and clinical data from three longitudinal registry trials (*N* = 3,668). SM C28:1, one of the biomarkers identified, showed an inverse relationship with incidence of CHD, but was not found to have a causal relationship by MR analysis (68).

However, definitive evidence for a causal role of circulating sphingolipids in HF, when assessed by MR analysis, remains elusive in the published literature. Possibly due to the contribution of the gut microbiome to circulating lipids and metabolites (71). Therefore, to identify causal factors by MR analysis, deeper genotyping that includes additional procedures for characterizing the microbiome may be required.

## Sphingolipids in animal models of heart failure

**Figure 3:**
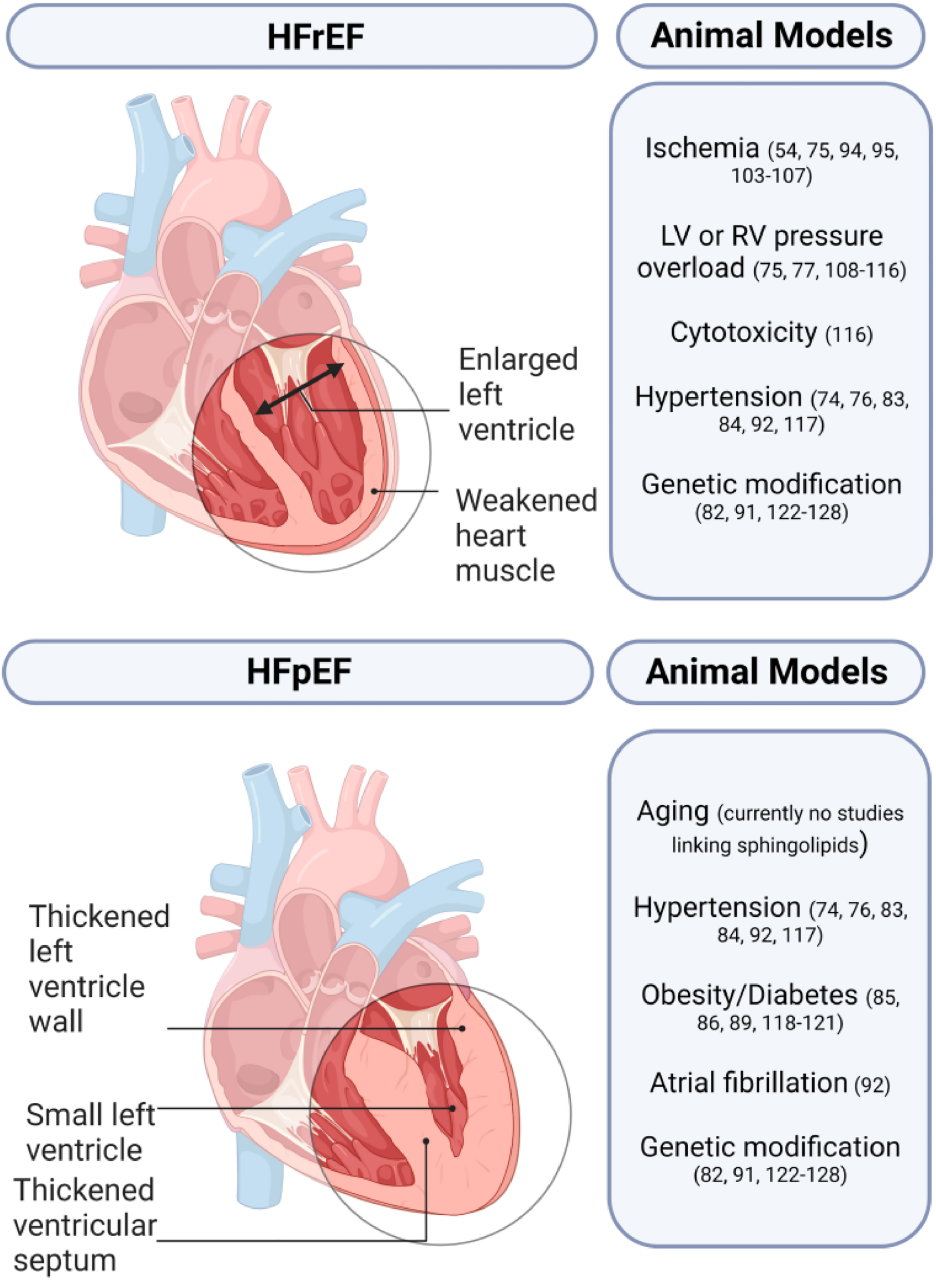
Sphingolipids in animal models of heart failure.

Animal models play a crucial role in studying HF and developing novel treatment strategies. Small animal models generally utilize pharmacological, surgical, and genetic modifications either alone or in combination while large animal models rely on surgical or pharmacological methods (Figure 3) (72, 73). A comprehensive list of animal models of HF with the respective sphingolipid alterations in blood serum, blood plasma, and/or myocardial tissue can be found in Table 3.

**Table 3:** Sphingolipids associated with animal models of heart failure.

Type	Animal Model	Sphingolipid Level(s)
Ischemia	LAD Ligation with or without genetic modification	Mouse • ↑Myocardial tissue *total stearoyl-SM* two weeks post-LAD compared to sham (74) • ↑Serum *total Cer*, C16:0, C18:0, C24:1, C24:0, and Cer C26:1 two weeks post-LAD; ↑Tissue *total Cer*, C14:0, C18:0, C20:0, C20:1, and Cer C22:1 two weeks post-LAD compared to sham (53) • =Serum *total Cer* 10 weeks post-LAD; ↑Myocardial tissue *total Cer*, C16:0, C24:1, and Cer C24:0 10 weeks post-LAD; ↓Myocardial tissue *total Cer*, C18:1, C18:0, C22:1, C22:0, and Cer C24:0 in cardiomyocyte-specific SPTLC2 KO mice 10 weeks post-LAD compared to sham and WT TAC (53) • ↑Myocardial tissue SM(OH)C14:1, SM C16:0, SM C16:1, SM C24:0, SM C24:1, and SM C26:0 in 12/15-LOX KO mice one-day post-LAD; ↓SM(OH) C24:1, SM C20:2, SM C26:0 in 12/15-LOX KO mice 8 weeks post-LAD compared to sham (75) • ↑S1P, =Sph, =DHS, =DHS1P in plasma and cardiac tissue; ↑Cer C16:0, C22:0, C24:0, and Cer C24:1, ↑C1P C18:0, C18:1, and C1P C22:0 from plasma in chronic serelaxin-treated mice 28 days post-LAD mice compared to vehicle (76) Rat • ↑Myocardial tissue Cer C16:0 post-LAD (77, 78) • =Myocardial mitochondria *total Cer* and *total SM* 4 weeks post-LAD (79) • ↑Myocardial mitochondria *total Cer*, =*total SM* in obese rats on HFD 4 weeks post-LAD (79) • ↑Myocardial tissue Cer d18:1/C24:0, SM d18:1/C16:0, SM d18:1/C24:1, SM d18:1/C24:2, SM d18:2/C16:0 one hour, one day and 10 days post-LAD compared to respective shams (80)
	Microembolization/Renal wrapping	Dog • ↑Myocardial tissue *total Sph* in the posterior wall of 8 h post-surgery compared to sham (81)
Pressure Overload	Aortic banding (AB)	Rabbit • ↓Myocardial tissue *total Sph* in AB neonatal rabbits treated with the ceramidase inhibitor *N*-oleoyl ethanolamine compared to AB untreated rabbits (82)
	Transverse aortic constriction (TAC) with or without genetic modification	Mouse • =Tissue *total erythro-sphingosylphosphorylcholine, Sph, Stearoyl SM,* and *total DHS* 1 day post-TAC compared to sham (74) • ↓Myocardial tissue *total erythro-sphingosylphosphorylcholine, Sph, Stearoyl SM,* and total *DHS* 1 week and 8 weeks post-TAC compared to respective sham (74) • ↑Myocardial tissue *total Cer*, C16:0, C18:0, and Cer C24:0; =Cer C20:0, and Cer C22:0 in CPT1b KO heterozygous mice 2 weeks after TAC, =*total Cer* and acyl chain Cers in WT TAC mice compared to sham 2 weeks post-TAC (83) • ↓Myocardial tissue *total SM* in TAC mice on HFD diet compared to TAC mice on CD 8 weeks post-TAC (84) • ↑Myocardial tissue *Asah1*, *Galc*, and *SGPP1* genes; ↓myocardial tissue *UGCG*, *SMS1*, *Acer2* genes in cardiomyocyte-specific Ppar*α* KO mice 2 weeks post-TAC compared to sham (85) • ↑S1P in coronary vascular perfusate from heart tissue in the Nogo-A/B KO mice 3 days post-TAC compared to sham (86) • ↑Myocardial tissue *total Cer*, C16:0, C24:1, and Cer C24:0; =Cer C18:1, C18:0, C20:0, and Cer C22:0 14 weeks post-TAC compared to sham (87) • ↑Tissue Cer C20:0, C22:0, C24:1, and Cer C24:0; =Cer C16:0, C18:1, and Cer C18:0 in ACSL1 OE mice 14 weeks post-TAC compared to sham (87) • ↑Tissue Cer C20:0, and Cer C22:0 Cer; ↓Cer C16:0; =Cer C18:1, C18:0, C24:1 and Cer C24:0 in cardiomyocyte-specific ACSL1 OE mice 14 weeks post-TAC compared to TAC (87) • ↓Myocardial tissue Cer C20:0, and Cer C22:0; =Cer C16:0, C18:1, C18:0, C24:1, and Cer C24:0 in cardiomyocyte-specific ACSL1 OE mice 14 weeks post-TAC compared to sham ACSL1 OE mice (88). Rat • ↓Myocardial tissue *total Sph* in mice on hypaconitine and glycyrrhetinic acid 5 weeks post-TAC acid compared to mice on CD 5 weeks post-TAC (89)
	Aortic constriction (AC) with or without HFD	Rabbit • ↓Myocardial tissue *total Cer*, C18:0, C20:0, C22:0, C24:0, and Cer C24:1; =Cer C16:0 21 days post AC compared to sham (90) • ↓Myocardial tissue *total Cer*, C18:0, C20:0, C22:0, C24:0, and Cer C24:1; =Cer C16:0 in rabbits given losartan 21 days post AC compared to sham (90) Rat • =Myocardial tissue Cer C16:0, C18:0, C20:0, and Cer C24:0 after 9 weeks of AC compared to control; ↓*total Cer* when compared to rats treated with DOX on HFD for 2 weeks (91)
Cytotoxic	Doxorubicin	Rat • ↑Myocardial tissue Cer C16:0 and C18:0 in rats treated with DOX on HFD for 2 weeks compared to control on western diet (91)
Atrial Fibrillation (AF)		Mouse • ↑Atrial tissue Cer C16:0 and GM3 C16:0 in 4 month aged mice with HF + AF (92)
Hypertension	Angiotensin II stimulation	Mouse • =Myocardial tissue *total Cer* levels in WT and non-HF cardiomyocyte-specific ANGII OE hearts and not modified by the 8 weeks of HFD; Similarly, =myocardial tissue *total Cer* in ANGII OE mice on CD; However, ↑myocardial tissue *total Cer* in ANGII OE mice with HF on HFD for 8 weeks compared with all other control groups (93) • ↑Myocardial tissue Cer d18:1/C20:0, d16:1/C23:0, d18:1/C19:0, d18:1/C22:0, d18:2/C18:1, and Cer d18:2/C20:1 in response to ANG II in both WT and cardiomyocyte specific DGAT1 OE mice. Basally, =Cer *d*18:1/C20:0, d16:1/C23:0, d18:1/C19:0, d18:1/C22:0, d18:2/C18:1, and Cer d18:2/C20:1 between the two mouse lines (92, 94)
	Dahl/SS	Mouse • =Myocardial tissue *total Cer* levels in WT and non-failing cardiomyocyte-specific ANGII OE mice hearts and not modified by 8 weeks on HFD; Similarly, =myocardial tissue *total Cer* in ANGII OE mice with HF on CD; However, ↑myocardial tissue *total Cer* in ANGII OE mice with HF on HFD for 8 weeks compared with all other control groups (93) Rat • =Myocardial tissue Cer C16:0 with rats on HFD and salt compared to rats on CD with salt (95)
	Spontaneously hypertensive Rat (SHR)	Rat • ↑Myocardial tissue Sph d18:1/C16:0, Sph d18:1/C18:0, Sph d18:0/C16:0, SM d18:1/C16:0, SM d18:1/C18:0, SM d18:1/C17:0, SM d17:1/C18:0, SM d19:1/C16:0, SM d18:1/C18:1, SM d18:2/C18:0, and DHS in SHR compared to control (96) • ↑Myocardial tissue Cer C16:0 in SHR at 12 weeks on HFD and high salt diet compared to rats at 12 weeks on low fat and salt, low fat and high salt, and in rats on low fat and high salt diets. Furthermore, ↑Myocardial tissue Cer C16:0 was observed in rats treated with the CPT1 inhibitor oxfenicine 12 weeks on low fat and high salt, and on HFD and salt diets (97)
T1D	Akita	Mouse • ↑Myocardial tissue *total Cer* in non-obese 3-month-old Akita mice compared to WT; ↓*total Cer* to WT levels when Akita mice were given insulin (98) • ↑Myocardial tissue *total Cer* in both WT and ATGL KO heterozygous 13- to 15-week-old mice after the induction of diabetes *via* high-dose 165 mg/kg STZ injections; Basally, *total Cer* was =in both sets of pre-diabetic mice (99) • ↓Myocardial tissue *total Cer* in cardiomyocyte-specific ATGL OE mice with and without high-dose 165 mg/kg STZ compared to control mice (99)
T2D	Db/db	Mouse • ↑Myocardial tissue *total Cer* at 12 weeks compared to WT; but ↓Myocardial *total Cer* at 15 weeks compared to WT (100)
	HFD	Mouse • ↑Myocardial tissue Cer d18:1/C14:0 in mice at 8 and 16-weeks on MFBD with cardiac hypertrophy compared to mice on lard-fat based diet and CD (101) • ↑Myocardial tissue d18 *total Cer*, d18:1/C18:0, d18:1/C18:1, d18:1/C22:0, and Cer d18:1/C24:0; ↑Myocardial tissue d16 *total Cer*, d16:1/C18:0, d16:1/C20:0, d16:1/C22:0, and Cer d16:1/C24:0 at 18 weeks on MFBD compared to mice on CD. ↓Cer species as above in mice on MFBD treated with myriocin compared to mice on MFBD alone (102) • =Myocardial tissue *total Cer* in mice on HFD and CD at 3 and 10 weeks (103) Rat • ↑Myocardial tissue Cer d18:1/C16:0 8 weeks on saturated HFD compared to rats 8 weeks on unsaturated HFD (104)
Genetic Modification	Genetic Modification	Mouse • ↑serum SM d18:1/C23:0, Cer d18:1/C22:0, Cer d18:1/C24:1, and Cer d18:1/C22:1 in 1 year aged GENA348 mice compared to WT (105)
	Overexpression	Mouse • ↑Myocardial tissue *total Cer* in mice with cardiomyocyte-specific OE of LPL^GPI^ compared to WT (106) • ↓Myocardial tissue *total Cer* in cardiomyocyte-specific OE of DGAT1 in mice after 2 weeks of intensive exercise compared to sedentary transgenic mice. ↓Myocardial tissue *total Cer* in mice with cardiomyocyte-specific OE of ACSL1 and DGAT1 compared to ACSL1 OE mice (107) • ↑Myocardial tissue *total Cer* in 4 months aged cardiomyocyte-specific PPAR*γ* OE mice compared to WT (108) • ↓Myocardial tissue *total Cer*, Cer C14:0, C16:0, C18:0, C18:1, C20:1, C24:0, and Cer C26:1 in mice with cardiomyocyte-specific OE of DGAT1 and PPARγ compared to mice only with cardiomyocyte-specific OE of PPARγ; =WT (109) • ↑Myocardial tissue *total Cer* in 18 day-old mice with cardiomyocyte specific OE of ACSL1 compared to control mice (110)
	Ablation/Knockdown	Mouse • ↑Myocardial tissue *total Cer*, C16:0, C18:0, C20:0, C20:1, C22:1, and Cer C24:1 with cardiomyocyte-specific DGAT1 KO compared to WT. DGAT1 dKO in heart and intestines showed ↓*total Cer*, C16:0, C18:0, C24:0, and Cer C24:1 compared to WT (111) • =Myocardial tissue *total Cer* in mice with whole body heterozygous Sptlc1 KO and with LpL^GPI^ transgenic mice compared to WT (106) • ↓Myocardial tissue *total Cer*, C18:0, C20:0, C24:0, and Cer C24:1, DHC, DHS, and Sph, but =S1P, SM C14:0, and Cer C16:0 in cardiomyocyte-specific Sptlc2 KO mice compared to WT (112) • ↑Myocardial tissue *total Cer* in heterozygous LCAD KO mice with 1 day fasting compared to 1 day fasted WT (113)

LAD, left anterior descending (of the coronary artery); Cer, Ceramide; Sph, Sphingosine; SM, Sphingomyelin; S1P, Sphingosine-1-phosphate; DHC, Dihydroceramide; DHS, Dihydrosphingosine; KO, knockout; OE, overexpression; CD, control diet; HFD, high fat diet; MFBD, milk-fat based diet; STZ, streptozotocin; ACSL1, acyl-CoA synthetase long chain family member 1; SPTLC2, serine palmitoyltransferase long chain base subunit 2; 12/15-LOX, arachidonate 15-lipoxygenase; Cpt1b, carnitine palmitoyltransferase 1B; *Asah1*, N-acylsphingosine amidohydrolase (acid ceramidase) 1; Galc, galactosylceramidase; and SGPP1, sphingosine-1-phosphate phosphatase 1; UGCG, UDP-glucose ceramide glucosyltransferase; SMS1, sphingomyelin Synthase 1; Acer2, alkaline ceramidase 2; PPAR, peroxisome proliferator activated receptor; ACSL1, long-chain acyl-CoA synthetase 1; DGAT1, diacylglycerol acyltransferase 1; LpL^GPI^, GPI-anchored lipoprotein lipase; **d** refers to 1,3 dihydroxy and is followed by **C** the number of carbons in each of the acyl side chains, The number of double bonds present is noted after the colon.

### Heart failure with preserved ejection fraction

HFpEF is a complex condition that only a few animal models have been able to replicate successfully. Animal models of HFpEF are typically pressure overload models as 55%–86% of patients with HFpEF have hypertension. The Dahl salt-sensitive (Dahl/SS) rat is the most popular HFpEF model and is characterized by hypersensitivity to sodium intake. Other popular models include animals with transverse aortic constriction (TAC), and gene knockout (KO), or overexpression (OE) that develop left ventricular hypertrophy (LVH).

A 2019 study observed an increase of the following sphingolipids prior to HF in Dahl/SS rats—1.27× N-palmitoyl-Sph C16:0, 1.67× glycosyl-N-stearoyl-Sph C18:0, 1.34× DHS, 1.72× N-palmitoyl-DHS C16:0, 1.24× palmitoyl SM C16:0, 1.28× stearoyl SM C18:0, 1.21× SM (d18:1/C17:0, d17:1/C18:0, d19:1/C16:0), 1.44× SM (d18:1/C18:1, d18:2/C18:0), and 1.32× Sph (96). Another study analyzed the myocardial sphingolipids at multiple timepoints in post-TAC mice compared to sham mice (74). Erythro-sphingosylphosphorylcholine was 1.04 at 1 day, 0.68 at 1 week and 0.69 at 8 weeks post-TAC. Sph and stearoyl SM levels decreased by half 8 weeks post-TAC. While DHS sharply increased at 1 day, then stayed relatively the same up to 8 weeks post-TAC (74). Another study determined that mice with HF could not adapt to excessive fatty acid supply vs. mice with LVH, both cohorts having angiotensin II (ANG II) OE (93). The hearts from HF mice on high fat diet (HFD), accumulated 50% more *total Cer*, aggravating contractile dysfunction, whereas the LVH mice on HFD showed a similar phenotype as the WT and no accumulation of *total Cer*, suggesting that impaired fatty acid oxidation in this model is associated with Cer lipotoxicity (93). CPT1b controls uptake of LCFAs in mitochondrial *β*-oxidation. Hearts from heterozygous CPT1b knockout (CPT1b^+/−^) mice subjected to TAC-induced pressure overload, showed substantially elevated *total Cer* levels driven by the Cer C16:0, C18:0, and C24:0 species, while sham WT and CPT1b^+/−^ mice showed no differences with respect to Cer content (83). While we cannot conclude that pressure overload is the sole driver of Cer accumulation, we can assume accumulated Cer in this model further aggravates the pressure overload induced hypertrophy caused by CPT1b deficiency.

Obesity and diabetes markedly increase the risk of HF, and alteration of sphingolipid metabolism contributes either directly or indirectly to metabolic stress in diabetes leading to diabetic cardiomyopathy and eventually HF (114–116). Diacylglycerol acyltransferase 1 (DGAT1) converts diacylglycerol (DAG) to triglyceride. Failing human hearts have severely reduced DGAT1 levels with concomitant accumulation of Cer and DAGs (117). Ablation of *Dgat* in mice led to no known cardiac dysfunction, and these mice express normal levels of circulating Cer. However, half of the cardiomyocyte-specific DGAT1 KO mice died within 9 months and their hearts showed an 85% and 95% increase in *total Cer* and DAGs, respectively, compared to littermate controls (111). Further studies are needed to delineate the role accumulated Cer, and accumulated DAGs play in causing HF followed by mortality in the cardiomyocyte-specific deletion of DGAT1. Another study found that decreased Cer d18:1/C20:0, increased Cer d16:1/C23:0, d18:1/C19:0, d18:1/C22:0, and d18:2/C20:1, were observed between control and DGAT1 OE animals, whereas 3% were responsive to ANG II administration. ANG II treatment in the OE mice resulted in a marked increase in heart size, systolic dysfunction, and cardiac fibrosis, with major reduction of the above-mentioned Cer, compared with control littermates (94). This could suggest Cer reduction renders cardiomyocytes more vulnerable to other pathological stresses.

HFD are the most popular metabolic-disease induced-HF model, generally considered beneficial in the setting of non-ischemic HF (97). Different types of HFD can be generated for rodents that mimic human diets with respect to the content of carbohydrate, protein, and saturated and unsaturated fatty acids, with relative ease and economical cost. Most HF patients in the human trials discussed above present with a form of diabetes and or obesity, making this model even more illuminating. A few studies from our lab showed that mice fed on a 60% milk-fat based diet (MFBD) as opposed to a 60% lard (oleate)-fat based diet and control diet (CD), had much higher levels of CerS2 derived Cer C:20 to C:26 in earlier stages of diabetes and diabetic cardiomyopathy. Further along the diet, the mice now presented with LV hypertrophy and CerS5 derived Cer C14:0 was significantly higher (101, 102). Thus, these diets give us the ability to identify specific sphingolipid species over the course of the events leading up to HF and in incident HF of animals. A new model of HFpEF uses metabolic and hypertensive stress elicited by HFD coupled with NOS inhibition and showed differentially methylated RNAs were enriched in sphingolipid metabolism (118, 119).

Akita mice (*Ins2*^Akita+/−^) have a mutation in the *Insulin2* gene and are a good non-obese type 1 diabetes mellitus (T1DM) model. At adulthood, the hearts of these mice had accumulated Cer C18:0 and exhibited preserved systolic function, reduced diastolic function, and increased inflammation. This lipotoxic cardiomyopathy phenotype was reversed with insulin replacement therapy (98). GENA348 mice, develop LVH and HF around 6 months of age (120). In 12-month GENA348 mice with systolic, diastolic dysfunction and LVH the following fold-change increases in serum samples were also observed—2.65× of SM C23:0, and a 2.26×, 1.80× and 1.45× of Cer C22:0, C24:1, and C22:1, respectively, compared to control mice (105).

A mouse model of atrial fibrillation (AF)-induced HF (cardiac-specific dnPI3K-Mst1 KO) showed significant increases of Cer C16:0 and the most abundant mammalian ganglioside, GM3 C16:0, in atria of AF + HF mice compared to control (92). These changes were also associated with increased atrial mass, though, treatment with the small molecule, a hydroximic acid derivative named BGP-15, reduced atrial mass and was positively correlated with reduced GM3 C16:0. Furthermore, BGP-15 treatment concomitantly increased insulin-like growth factor 1 binding to caveolins, a cardioprotective signaling pathway, thought to be inhibited by GM3 (92). This study suggests GM3 C16:0 may contribute to atrial pathology in the context of HF.

### Heart failure with reduced ejection fraction

Most HF animal models are used in research to study HFrEF due to the clear clinical diagnostic criteria, and parameters. The two main animal models for HFrEF are ischemia/infarction and pacing models (72). Other less common models include surgically induced mitral regurgitation, arteriovenous fistula creation, and administration of doxorubicin (72, 121).

In the ischemia/infarction, microsphere beads are injected intracoronarily down selected arteries under fluoroscopy or there is temporary or permanent occlusion of the left anterior descending (LAD) coronary artery. A study on this model found that stearoyl-SM was reduced by less than 50% in heart tissue 5 days post permanent-LAD ligation in mice compared to mice with sham surgery (74). Rats with HF induced *via* permanent LAD ligation showed higher Cer C16:0 in the heart tissue compared to sham-operated mice (77). 8 weeks post MI, these rats went on a 45% kcal saturated and unsaturated combination HFD but showed no further exacerbation of LVH and no further increase in tissue *total Cer*. Thus, suggesting Cer myocardial content is not dependent on availability of fatty acids in the context of the failing heart. Another study also showed a similar increase in *total Cer* content after MI, interestingly, once the rats were placed on a 60% kcal high-saturated fat diet, unlike the previous study, the myocardial *total Cer* content increased, though there was no further progression of HF (78, 122). Isolated hearts from male rats subject to ischemia led to 14.1% increase in myocardial *total Cer*, 48.4% increase with ischemic reperfusion (IR), and partial reversal with ischemic preconditioning. The most significant being that of Cer C16:0, C18:0, C18:1, C18:2, C20:4, C22:5, and Cer C22:6 (123). These studies have shown that increased release of MCFA, LCFA, and VLCFA of Cer into circulation and found within cardiac tissue are often associated with negative effects on cardiac function. Although it is essential to develop pharmacological inhibitors of specific CerS, there have been multiple roadblocks in this process.

## Conclusions

Great strides have been made in understanding the sphingolipidome in patients with HF. The Mayo Clinic's CERAM panel's ratios of the VLCFA to MCFA Cer are becoming increasingly popular for assessing the risk of HF development and progression. Evidence suggests these novel Cer biomarkers will continue be more successful than conventional risk factors in predicting adverse cardiac events and the onset of HF, especially when applied on a larger scale. However, there is still no clear evidence to show mechanistically how Cer C24:0 is linked to beneficial pathways or Cer C16:0 to adverse pathways in HF. More studies are needed to determine how Cer (and other sphingolipids) with different acyl chain lengths regulate various signaling pathways. Despite these developments, prognosis for HFrEF and HFpEF patients remain poor. Thus, when considering future studies in humans and animal models of HF some points should be considered.

The first point to consider: different results can be obtained from different materials analyzed, and while tissue sphingolipid levels are useful in patients receiving non-pharmacological intervention such as LVAD, it is of more clinical benefit to discover potent biomarkers in circulation, i.e., serum or plasma. This is due to ease of accessibility, and ability to collect samples more frequently in follow-up appointments. Nonetheless, tissue sphingolipid levels are important In fact, tissue from HF patients prior to LVAD showed induction of SPTLC3 at the protein level, a follow-up showed reduced SPTLC3 levels post-VAD (53).

Which leads to the second point for consideration, it is becoming more apparent that the *de novo* sphingolipid synthesis pathway can make very diverse sphingolipids in turn triggering unique signaling cascades. While d18 backbone sphingolipids are the most abundant and well-studied, there is mounting evidence to suggest substrate availability is the major determinant for the type of sphingolipids synthesized. For example, variable amino acid substitutes for serine, such as alanine or glycine, result in loss of the hydroxyl group at the alpha carbon to produce deoxysphingolipids. Deoxysphingolipids have been shown to play important roles in pathogenesis of both T1 and T2D. SPTLC3 can use isoleucine, a branched chain amino acid, to create methyl-branched long chain based sphingolipids, the implications of these in HF have yet to be elucidated (124). Though, it was recently revealed that transgenic mice with pressure-overload induced HFrEF showed impaired catabolism of myocardial branched-chain amino acids, upon which restoration of these amino acids reversed the dysfunction in this HFrEF animal model (125). Another way substrate availability can determine which sphingolipids to synthesize is availability of myristoyl- or stearoyl-CoA over palmitoyl-CoA. SPTLC3 can readily use myristoyl-CoA in place of palmitoyl CoA (25). Thus, more recent evidence points towards less abundant sphingolipid species with d16, d19 and d20 backbones associated with events leading up to and in HF (27, 30). A few animal studies highlighted in this review observed significant differences of SPTLC3-derived sphingolipid species. This could provide a new avenue of research in developing specific inhibitors of CerS and SMS.

The third point to consider: Which sphingolipidomic analyses technique to use. The omics field has seen a rapid increase in both the number of studies and the size of datasets. In parallel, liquid chromatography coupled with mass spectrometry (LC-MS) approaches have advanced yielding thousands of distinct MS peaks representing individual sphingolipid species and their metabolites (126, 127). Targeted LC-MS measurements are more sensitive, accurate, and quantitative than untargeted ones. However, most studies on human cohorts only focus on d18 Cer or SM species, and it would be of more beneficial consequence to include targeted LC-MS runs for d16, d19 and d20 backboned sphingolipids with acyl chains ranging from C14 to C26, as well as other species such as Sph, S1P, DHS1P, monohexosylceramides (MHC), and the glucosylceramides. While using targeted LC-MS may overlook associations with many known and unknown species in HF, untargeted mass spectrometric techniques can detect species that were not considered in targeted runs and associate them with HF (128). Though untargeted techniques are still being fine-tuned and require more starting sample per run. It is likely that multiple sphingolipids unique to each HF group (HFrEF, HFpEF, and HFbEF/HFmrEF) are involved in different pathogenesis, and these sphingolipid signatures could provide more concise cut-off values for classifying patients.
